# Gender Differences in the Behavioral Symptom Severity of Prader-Willi Syndrome

**DOI:** 10.1155/2015/294127

**Published:** 2015-11-08

**Authors:** Masao Gito, Hiroshi Ihara, Hiroyuki Ogata, Masayuki Sayama, Nobuyuki Murakami, Toshiro Nagai, Tadayuki Ayabe, Yuji Oto, Kazutaka Shimoda

**Affiliations:** ^1^Department of Psychiatry, Dokkyo Medical University Koshigaya Hospital, 2-1-50 Minami-Koshigaya, Koshigaya, Saitama 343-8555, Japan; ^2^Ikezawa Hospital, 551 Shimo-Shingo, Hanyu, Saitama 348-0046, Japan; ^3^Department of Psychiatry, Dokkyo Medical University School of Medicine, 880 Kita-Kobayashi, Mibu-Machi, Shimotuka-Gun, Tochigi 321-0293, Japan; ^4^Department of Pediatrics, Dokkyo Medical University Koshigaya Hospital, 2-1-50 Minami-Koshigaya, Koshigaya, Saitama 343-8555, Japan; ^5^Nakagawanosato Ryoiku Center, 222 Shimo-Akaiwa, Matsubushi-Machi, Kita-Katsushika-Gun, Saitama 343-0116, Japan

## Abstract

*Objectives*. This study measured gender differences in Prader-Willi syndrome (PWS) in regard to the severity of behavioral symptoms. *Methods*. The Food Related Problem Questionnaire (FRPQ), the Aberrant Behavior Checklist Japanese Version, the Childhood Routines Inventory, the Pervasive Developmental Disorders Autism Society Japan Rating Scale, and Japanese ADHD-RS were administered to PWS patients (45 males aged 6 to 58 and 37 females aged 6 to 45). To examine the effects that gender and genotype have on the severity of each symptom, two-way ANOVAs were conducted. *Results*. Significant interactions were found only in regard to FRPQ scores, such as FRPQ total score (*F*(1, 78) = 8.43, *p* < 0.01). The FRPQ of male deletion (DEL) individuals was higher than that of female DEL and male mUPD. The FRPQ of male maternal uniparental disomy (mUPD) was lower than that of female mUPD. *Conclusions*. In terms of problem behaviors, routines, autistic behaviors, and hyperactivity, no significant differences were found. Food-related behaviors in DEL were more severe in males, although those in mUPD were less severe in males.

## 1. Introduction

Prader-Willi syndrome (PWS) is a genetic disorder caused by a loss of expression of the paternally derived genes on chromosome 15q11-13. The causes of this disruption include paternal deletion (DEL) of 15q11-13 and maternal uniparental disomy 15 (mUPD, when both copies of chromosome 15 are maternally inherited) [1]. As a neurodevelopmental disorder, PWS is associated with neonatal hypotonia, hypogonadism, hyperphagia, progressive obesity, and mild to moderate mental retardation [2]. The physical manifestations of PWS include short stature, small hands and feet, hypopigmentation, and craniofacial anomalies. Based on epidemiological surveys, the birth rate is estimated at around 1 in 25,000 [3].

The behavioral manifestations of this syndrome include hyperphagia [4, 5], temper tantrums [6], obsessive-compulsive behaviors [7, 8], repetitive and ritualistic behavior [9], self-injurious behavior [10, 11], autistic behaviors [12, 13], hyperactive/impulsive behaviors [14, 15], and psychiatric disorders [16]. Due to lack of specific drugs in controlling PWS-related behaviors, pharmacological treatment should only be used with caution and in combination with behavioral management and psychiatric support [17].

In terms of population prevalence for people with Prader-Willi syndrome, the gender ratio is close to 1 : 1 [1, 18–20], including non-Western countries [21]. So far, however, there has been little research into gender differences in individuals with PWS in relation to behavioral symptom severity. One piece of data was presented by Dykens [22], who found that females are more inclined to pick their skin than males. Such gender difference in terms of skin picking was also found in individuals with nonspecific mental retardation [23]. When food-related behaviors were analyzed by both gender and genotype, female mUPD patients were found to be less severely affected than female DEL patients in terms of length of gavage feeding and a later onset of hyperphagia. This difference between mUPD and DEL was not found in male patients [24].

Aside from Prader-Willi syndrome, certain mental disorders show marked gender differences in the diagnosis rates. For example, unipolar depression is twice as common in women. In contrast, the prevalence of alcohol dependence is more than twice as high in men than in women [25]. The diagnosis of antisocial personality disorder is more than three times as high in men than in women. On the other hand, severe mental disorders such as schizophrenia and bipolar disorder are associated with no pronounced gender differences in terms of the prevalence rate [25]. Among neurodevelopmental disorders, autism spectrum disorders (ASD) have consistent male predominance ranging from 2.5 : 1 to 4 : 1 in individuals with autistic disorder and 9 : 1 in individuals with Asperger disorder [26–28]. Attention deficit hyperactivity disorder (ADHD) is more commonly diagnosed in males than females, with male-to-female ratios ranging from 4 : 1 to 9 : 1 [29]. Eating disorders, which are one of the most important psychiatric categories of the behavioral aspects of PWS, are more common among females than males in both anorexia nervosa and bulimia nervosa [30, 31]. Equally important is the fact that the reverse pattern of gender disparity is found in “subthreshold binge eating disorder” (0.6% women and 1.9% men) and that roughly comparable gender distribution is observed in the prevalence of “any binge eating” in women (4.9%) and men (4.0%) [32].

In terms of problems behavior in individuals with non-PWS mental retardation, findings regarding gender differences in Williams syndrome have been divided. Leyfer et al. [33], Pérez-García et al. [34], and Klein-Tasman et al. [35] did not find gender differences in psychiatric and problem behaviors. However, Porter et al. [36] found greater externalizing, somatic, affective, and conduct problems in girls. Also, Dykens [37] found that fearfulness was higher for girls than for boys. In the Down syndrome sample, problem behavior was more prevalent in boys than in girls [38]. By contrast, psychosis was predominantly seen in females with Down syndrome [39].

This study aims to explore gender differences in PWS in regard to the severity of behavioral symptoms. There are three inherent advantages in this study. Firstly, this is the only study of its kind in regard to gender differences in behavioral aspects of PWS, based on a large sample of the rare genetic disorder. Secondly, all subjects were recruited from a single institution and confirmed genetically with PWS using fluorescence in situ hybridization or the methylation test. Thirdly, all subjects with PWS were assessed by a single clinical psychologist (H.O.). Hence, the psychometrical data of this study can avoid the risk of interrater variability caused by participants being assessed by multiple assessors. At the same time, it should be noted that this single-center study has not only strength, but also weakness, because of sample selection bias and a small sample size.

## 2. Subjects and Methods

This study started upon receiving approval from the ethics committee of Dokkyo Medical University Koshigaya Hospital with which the authors were affiliated. After obtaining informed consent, the neurocognitive and behavioral assessment of each participant was carried out.

### 2.1. Subjects

Participants were 82 Japanese individuals with PWS recruited from a single location. The Department of Pediatrics, Dokkyo Medical University Koshigaya Hospital, was used for this purpose. All patients were diagnosed with PWS using fluorescence in situ hybridization or the methylation test. The participants consisted of 45 males (aged 6 to 58) and 37 females (aged 6 to 45), including 34 males and 25 females confirmed as having DEL involving 15q11-13 and 11 males and 12 females confirmed as having mUPD of chromosome 15 (Table 1). Psychotropic medications were prescribed to 16 out of 45 males and 9 out of 37 females.

### 2.2. Methods

*The Assessment of Behavior*. An extended battery of behavioral assessment was employed. In all cases, the psychologist (H.O.) involved in collecting data was blind to the genetic status of each patient.

### 2.3. Measures

#### 2.3.1. Intellectual Ability

To measure intellectual ability, a Japanese version of the Wechsler Intelligence Scale [40–43] was administered.

#### 2.3.2. Food-Related Behaviors

To assess the severity of food-related behaviors, the Food Related Problem Questionnaire (FRPQ) was administered. This is an informant-based questionnaire to assess eating behaviors in people with PWS, consisting of 16 items, with three subscales (preoccupation with food (P), impairment of satiety (S), and other food-related negative behaviors (N)). Examples of the questions are as follows: “How often does the person compare the size or content of their meal with others?” (P); “After a normal sized meal, how often does the person say they still feel hungry?” (S); and “If given the opportunity, how often would the person “help themselves” to food which they should not have?” (N). As Russell and Oliver [44] presented, the FRPQ has sufficiently robust psychometric properties to appraise the food-related problems in individuals with PWS.

#### 2.3.3. Aberrant Behaviors

To assess the degree of problem behaviors in individuals with PWS, the Aberrant Behavior Checklist Japanese Version (ABC-J) [45] was applied. It is a 58-item checklist which takes about 10–15 minutes to complete. There are five subscales: (a) irritability and agitation, (b) lethargy and social withdrawal, (c) stereotypic behavior, (d) hyperactivity and noncompliance, and (e) inappropriate speech. It was found that the ABC identifies salient features of mental illness in individuals with mental retardation [46], including autism spectrum disorder [47], and is an effective tool in measuring treatment response [46, 48].

#### 2.3.4. Routine Behaviors

To measure the extent of routines, the Childhood Routines Inventory (CRI) was administered. This is a parent-report checklist of commonly occurring children's routines [49], consisting of 19 items, including 5 items for “just right behavior” and 5 items for “repetitive behavior.” An example of the former is “Prefers to have things done in a particular order” and that of the latter is “Prefers the same household schedules or routines.” It was found that the CRI is an effective tool in measuring the severity of routine behaviors in normal young children [49] and children with Down syndrome [50].

#### 2.3.5. Autistic Symptomatology

Autistic symptomatology was assessed using the Pervasive Developmental Disorders Autism Society Japan Rating Scale (PARS) [51, 52]. This scale is a behavior checklist, developed as a screening questionnaire to determine pervasive developmental disorders (PDDs). When assessing adolescents and adults, 33 items for adolescents, partially shared by those for children, are applied for the evaluation of current autistic states. The PARS for adolescents is made up to five clinical subscores consisting of interpersonal skills (6 items), communication (7 items), obsession (6 items), problematic behaviors (11 items), and hypersensitivity (3 items).

#### 2.3.6. Inattention and Hyperactivity/Impulsivity

The Japanese ADHD-RS [53] was administered to all participants. The ADHD-RS [54] obtains parent ratings regarding the frequency of each ADHD symptom based on DSM-IV criteria. The scale consists of 2 subscales: inattention (9 items) and hyperactivity/impulsivity (9 items). Parents are asked to state the degree to which they best describe the child's behavior over the previous 6 months. All items are scored on a 4-point Likert scale from 0 (“rarely or never”) to 3 (“always or very often”), with higher scores reflecting higher degree of inattention and hyperactivity/impulsivity. The reliability and the validity of the Japanese ADHD-RS have already been established [53].

By means of a numerical coding system, all data were guarded under strict confidentiality and anonymity. The data were analyzed by SPSS 20.0J for Windows. The results are expressed as mean (SD and range). To examine the effect that gender and genotype have on the severity of behavioral symptoms, two-way analyses of variance (ANOVAs) were conducted. The two gender groups (male versus female) and the two genotypes of PWS (DEL and mUPD) were used as independent variables, and the behavioral scores were used as dependent variables.

## 3. Results

As Table 1 shows, the mean IQs in both males and females were slightly less than 50, with no statistical difference between both groups. These scores are more than 50 points under the normative population score of 100, indicating a considerable impairment in intellectual abilities. As far as genotypes are concerned, a statistically significant difference was found between DEL and mUPD, with higher scores in the DEL group [15, 55].

Table 2 shows the results in regard to the degree of behavioral symptoms of PWS individuals. In order to examine the effects that genders and genotypes have on the symptom's severity, two-way ANOVAs were conducted. The two gender groups and the two genotypes of PWS were used as independent variables, and the scores of the FRPQ, the ABC-J, the CRI, the PARS, and the ADHD-RS were used as dependent variables.

Statistically significant interactions between genders and genotypes were found only in regard to FRPQ scores: FRPQ total score (*F*(1,78) = 8.43, *p* < 0.01), FRPQ-P (*F*(1,78) = 6.66, *p* < 0.05), FRPQ-S (*F*(1,78) = 7.74, *p* < 0.01), and FRPQ-N (*F*(1,78) = 4.04, *p* < 0.05). On finding this, a Bonferroni procedure was performed to test the simple main effects.

In terms of the FRPQ total score, it was found that the score of male DEL was higher than that of female DEL (*F*(1,78) = 4.22, *p* < 0.05); on the contrary, the score of male mUPD was lower than that of female mUPD (*F*(1,78) = 4.56, *p* < 0.05). Besides, the FRPQ score of male DEL was found to be higher than that of male mUPD (*F*(1,78) = 17.43, *p* < 0.01).

**Figure 1:**
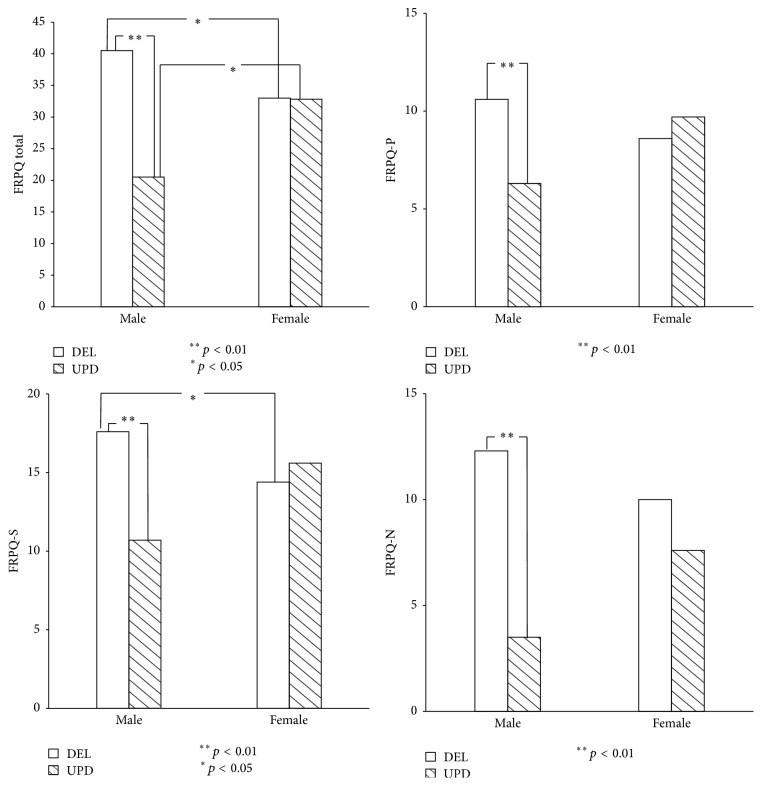
The effect of gender (male versus female) and genotype (DEL versus UPD) of PWS on the total score and preoccupation (P), impairment of satiety (S), and composite negative behavior (N) domains of FRPQ.

In regard to the FRPQ subscores, the FRPQ-P (preoccupation) score of male DEL was higher than that of male mUPD (*F*(1,78) = 8.79, *p* < 0.01). The FRPQ-S (satiety) score of male DEL was higher than that of female DEL (*F*(1,78) = 4.43, *p* < 0.05) and was higher than that of male mUPD (*F*(1,78) = 11.32, *p* < 0.01). The FRPQ-N (negativity) score of male DEL was higher than that of male mUPD (*F*(1,78) = 15.84, *p* < 0.01) (Figure 1).

## 4. Discussion

To our knowledge, this study is the first attempt to compare a wide range of behavioral features of PWS between male and female groups. To examine the effect that genotypes as well as genders have on the severity of behavioral symptoms, two-way analyses of variance (ANOVAs) were conducted. Two gender groups and two genotypes of PWS were used as independent variables. Apart from food-related behaviors, no significant statistical differences were found between male and female in regard to other behavioral features, such as problem behaviors, routines, autistic symptoms, inattention, and hyperactivity. As a whole, male and female PWS patients seem to be more similar than different regarding PWS-related behavioral symptoms. In this respect, our data accord with previous reports, which found similarities, rather than differences, in behavior between male and female [56–58].

The two-way ANOVAs to examine the interaction between gender and genotype, followed by the Bonferroni procedure to test the simple main effects, showed the following: food-related behaviors of male DEL were more severe than those of female DEL, and on the contrary food-related behaviors of male mUPD were less severe than those of female mUPD. Thus, the reverse pattern of gender disparity between the two genotypes was found.

These data contradicted the recent report of Jauregi et al. [58], who found female dominant scores in only two among the twenty-two items of the Developmental Behavior Checklist for Adults: “irritability” and “distress over small changes in their routine or environment.” Aside from these items, they did not find significant differences between male and female behaviors. At the same time, they found significant differences between DEL and non-DEL, such as “increase in appetite” with a higher score in DEL. Indeed, Dykens et al. [59] showed the lack of significant relations between food-related behaviors and genetic status. However, as the results suggest, the relationship between hyperphagia and genotype may be complicated by gender disparity. There seems to be a need for further investigation in terms of the impact of the gender differences on the relationship between genetic status, hyperphagia, and other behavioral symptoms.

It is evident that further methodological limitations exist in this study. First, the impact of biological changes in chronological adolescence was not considered. Unfortunately, the number of patients enrolled in this study was too small to analyze gender differences in the behavioral data in multiple age groups. According to Ogata et al. [15], there is a growing tendency for the autistic and impulsive behavioral problems, which are more severe in mUPD than in DEL that can manifest themselves later in adolescence. Likewise, gender differences in the behavioral symptom severity should take age, as well as genotypes, into consideration.

Second, this study did not encompass the entire range of psychiatric disorders relevant to gender differences in PWS. For example, it remains to be seen in regard to clinical categories whose rate is known to show striking gender differences. They include male-dominant disorders, such as alcohol dependence and antisocial personality disorder, and female-dominant ones, such as depression, anxiety, and somatic complaints [25]. Moreover, this study did not cover affective and psychotic disorders, in spite of the fact that the PWS group was known to have higher rates of affective disorders with psychotic features [60]. Although little is known about gender differences, PWS individuals with a psychotic disorder showed a disproportionate number of mUPD patients [61]. A more comprehensive study is required to illuminate gender differences in the severity of various psychiatric categories in PWS.

Third, a future study should assess the influence of endocrinological factors including growth hormone therapy and diabetes on the behavioral aspects with PWS focusing on gender differences. Based on 35 Brazilian patients with PWS, Quaio et al. [62] demonstrated that growth hormone treatment considerably improved the control of weight gain and body mass index for female patients but no effect on either parameter in male patients. They suggested that in male patients the benefits of growth hormone treatment may have been overcome by other factors, such as food-intake behaviors. The relationship between the gender differences in the effects of growth hormone treatment and those in food-related behaviors is an area worthy of further exploration, because it could potentially throw a new light on the possibility of gender-specific hormonal treatment to PWS. Aside from food-related behaviors, another gender-specific hormonal treatment was conducted by Kido et al. [63]. They found that testosterone replacement therapy improved secondary sexual characteristics and body composition without adverse behavioral problems in male patients with PWS. Finally, due to a single-institution study that aimed at a rare genetic disorder, the size of sample is relatively small. For this reason, the male-female similarity regarding PWS-related behaviors in this study should be interpreted with caution.

## Figures and Tables

**Table 1:** Patient characteristics.

	Total	Male	Female	DEL	mUPD	*p* value (*t*-test)	
						Gender groups	Genotype groups
Number (patients)	82	45	37	59	23		
IQ (mean ± SD)	49.3 ± 9.6	49.9 ± 9.3	48.2 ± 10.3	49.7 ± 9.8	44.6 ± 6.5	0.44	0.001^*∗*^
IQ range	39–84	39–79	39–84	39–84	39–62		
Age (mean ± SD)	18.6 ± 9.4	19.7 ± 10.2	16.9 ± 8.0	19.0 ± 9.5	15.4 ± 7.6	0.11	0.017^*∗*^
Age range	6–58	6–58	6–45	6–58	6–36		

^*∗*^ *p* < 0.05.

**Table 2:** The FRPQ, ABCJ, CRI, PARS, and ADHD-RS scores and the results of two-way ANOVA using the two gender groups and the two genotypes.

	Total	Gender		Genotype		ANOVA interaction	
		Male	Female	DEL	mUPD	*F*	*p*
FRPQ total	34.4 ± 15.0	35.6 ± 15.3	33.0 ± 14.8	37.3 ± 14.6	27.0 ± 13.6	8.43	0.005^*∗*^
FRPQ-P	9.3 ± 4.4	9.5 ± 4.5	9.0 ± 4.3	9.8 ± 4.4	8.0 ± 4.2	6.66	0.012^*∗*^
FRPQ-S	15.4 ± 6.3	16.0 ± 6.1	14.8 ± 6.5	16.3 ± 6.1	13.3 ± 6.3	7.74	0.007^*∗*^
FRPQ-N	9.7 ± 6.9	10.1 ± 7.1	9.2 ± 6.7	11.3 ± 6.8	5.7 ± 5.3	4.04	0.048^*∗*^

ABCJ total	33.2 ± 29.4	34.3 ± 30.8	31.8 ± 27.9	30.7 ± 27.0	39.8 ± 35.2	0.91	0.344
ABCJ excitement	11.3 ± 10.2	12.0 ± 10.2	10.4 ± 10.2	11.2 ± 10.1	11.6 ± 0.6	1.20	0.277
ABCJ apathy	7.1 ± 8.0	6.6 ± 7.3	7.7 ± 8.9	5.8 ± 6.3	10.6 ± 10.7	2.16	0.145
ABCJ stereotype	2.1 ± 3.2	1.9 ± 2.9	2.3 ± 3.6	1.7 ± 2.7	3.2 ± 4.2	0.93	0.337
ABCJ hyperactivity	8.6 ± 9.2	9.5 ± 10.6	7.4 ± 7.1	8.1 ± 8.7	9.9 ± 10.7	0.06	0.812
ABCJ inappropriate	4.0 ± 3.2	4.2 ± 3.2	3.8 ± 3.3	4.0 ± 3.3	4.2 ± 3.0	0.27	0.603

CRI total	3.1 ± 1.6	2.8 ± 1.5	3.3 ± 1.8	3.1 ± 1.7	2.9 ± 1.6	0.38	0.542
CRI Fre	9.3 ± 5.8	8.0 ± 5.1	10.7 ± 6.2	9.7 ± 5.8	8.5 ± 5.8	0.84	0.364
CRI Jr	0.8 ± 0.8	0.8 ± 0.9	0.7 ± 0.8	0.9 ± 0.7	0.5 ± 1.0	0.85	0.363
CRI JrFre	2.1 ± 2.6	2.2 ± 2.8	2.1 ± 2.5	2.3 ± 2.0	1.7 ± 3.6	0.51	0.480
CRI Re	0.7 ± 0.6	0.6 ± 0.6	0.8 ± 0.7	0.7 ± 0.7	0.8 ± 0.6	0.16	0.687
CRI ReFre	2.1 ± 2.1	1.5 ± 1.5	2.7 ± 2.5	2.1 ± 2.3	2.2 ± 1.8	0.03	0.868

PARS child	9.8 ± 7.0	10.2 ± 7.7	9.2 ± 5.5	9.4 ± 6.6	11.3 ± 8.0	0.00	0.972
PARS adolescent and adult	17.0 ± 9.2	16.9 ± 9.4	17.2 ± 9.2	15.9 ± 9.1	21.6 ± 8.8	1.06	0.307

ADHD-RS total	4.9 ± 5.9	5.3 ± 6.7	4.1 ± 3.8	4.6 ± 5.7	5.7 ± 6.6	0.02	0.902
ADHD-RS inattention	2.9 ± 3.4	3.1 ± 3.8	2.5 ± 2.3	2.7 ± 3.0	3.5 ± 4.3	0.02	0.877
ADHD-RS hyperactivity/impulsivity	2.0 ± 3.1	2.2 ± 3.5	1.5 ± 2.2	1.9 ± 3.3	2.3 ± 2.5	0.16	0.687

^*∗*^ *p* < 0.05.

FRPQ: Food Related Problem Questionnaire; FRPQ-P: preoccupation with food, S: impairment of satiety, N: composite negative behavior; ABC-J: Aberrant Behavior Checklist-Community Japan Rating Scale; CRI: Childhood Routine Inventory; CRI Jr: just right behaviors, Re: repetitive behaviors, Fre: frequency/intensity score; PARS: Pervasive Developmental Disorders Autism Society Japan Rating Scale; ADHD-RS: ADHD Rating Scale.
